# Supplementary material for: The decay of the refocused Hahn echo in double electron–electron resonance (DEER) experiments
Source: Magn Reson (Gott). 2021 Apr 16;2(1):161–73. doi: 10.5194/mr-2-161-2021 (PMC10539729; doi:10.5194/mr-2-161-2021)
Supplement: The supplement related to this article is available online at: https://doi.org/10.5194/mr-2-161-2021-supplement. [file mr-2-161-supplement.pdf]

Supplement of Magn. Reson., 2, 161–173, 2021
https://doi.org/10.5194/mr-2-161-2021-supplement
© Author(s) 2021. CC BY 4.0 License.

Supplement of

The decay of the refocused Hahn echo in double electron–electron resonance (DEER) experiments

Thorsten Bahrenberg et al.

Correspondence to: Stefan Stoll (stst@uw.edu) and Daniella Goldfarb (daniella.goldfarb@weizmann.ac.il)

The copyright of individual parts of the supplement might differ from the article licence.

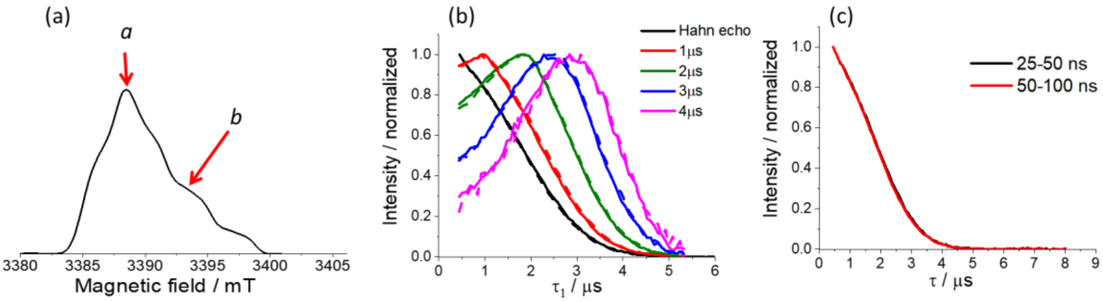

Figure S1. (a) W-band echo-detected EPR spectrum of 100 μM 3-maleimido-proxyl in H_2O/glycerol (80:20 v/v) measured at 25 K (different batch than that shown in Fig. 3). (b) Normalized echo intensity as a function $\boldsymbol{\tau_1}$ of for several $\boldsymbol{\tau_2}$values (indicated on the Figure) measured at the two different field positions, *a* (solid lines) and *b* (dashed lines) as indicated in (a), together with a Hahn echo decay. The π/2 and π pulse durations were 25 and 50 ns. (c) Comparison of the Hahn echo decay recorded at position *a* with different π/2 and π pulses as noted on the figure.

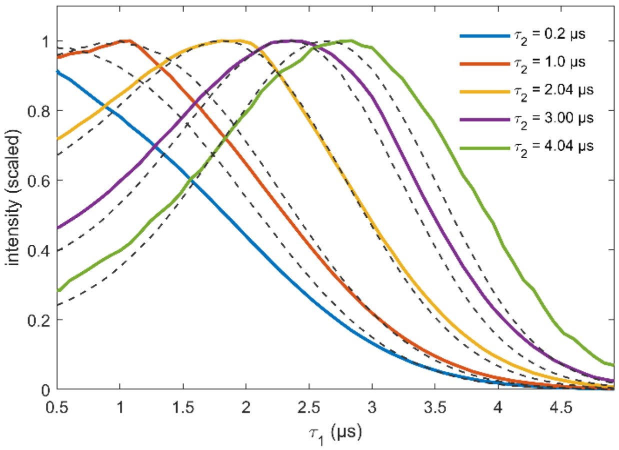

Figure S2. Comparison of the experimental refocused Hahn echo decays for 3-maleimido-proxyl (solid coloured lines; as in Figure 3c) with the CCE simulations (dashed black lines; as in Fig. 9b). The simulations reproduce the experimental trends well. The largest deviations are seen at short and large τ_2 values.

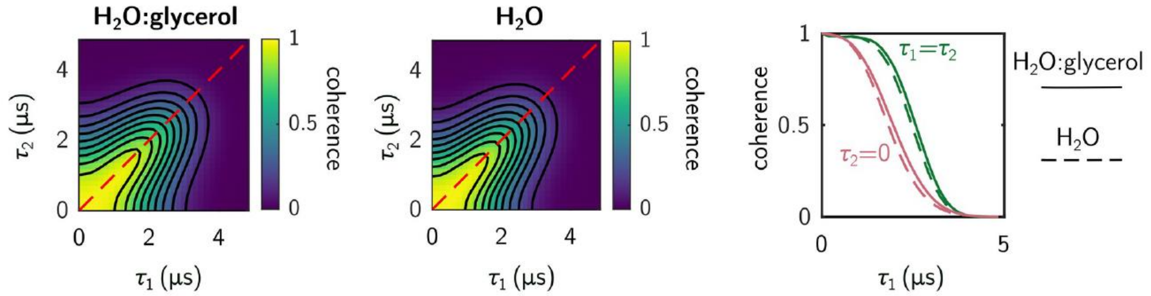

Figure S3. CCE simulations of the refocused echo decay for different solvents: H_2O:glycerol (left) and H_2O (centre), and a comparison of the $\tau_2 = 0$ and $\tau_1 = \tau_2$ traces (right). The H_2O:glycerol simulation is the same 3-CCE simulation as in Figure 10. The H_2O simulation has an identical input, save for the solvent environment, a pure water solvent instead of a mixture of water and glycerol. Consequently, the water-only system has 494 one-clusters, 4,124 two-clusters, and 49,178 three-clusters. Both solvents give very similar decays.

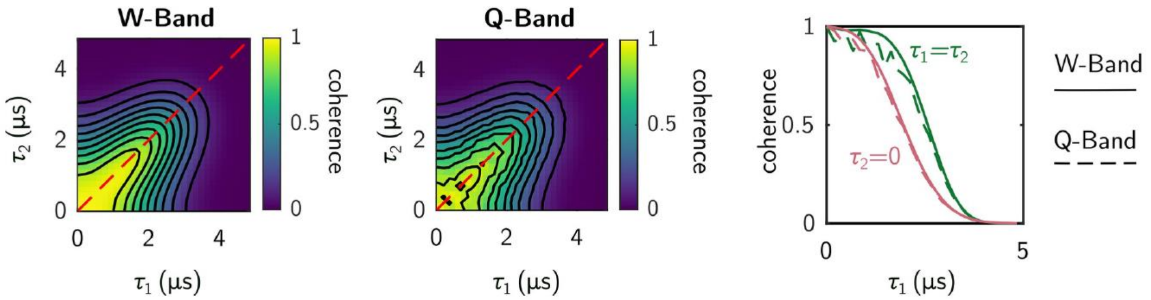

Figure S4. CCE simulations of the refocused Hahn echo for W-band (left) and Q-band (right), and a comparison of the $\tau_2 = 0$ and $\tau_1 = \tau_2$ traces (right). The W-Band simulation is the same 3-CCE simulation as in Figure 10. The Q-Band simulation has an identical input, except for the applied magnetic field; B_0 = 1.2 T. While there are more ESEEM modulations in the Q-Band simulation, the decay envelopes are close to identical.
